# Heart inflammation and piscine orthoreovirus genotype-1 in Pacific Canada Atlantic salmon net-pen farms: 2016–2019

**DOI:** 10.1186/s12917-022-03409-y

**Published:** 2022-08-10

**Authors:** Mark P. Polinski, Lynden A. Gross, Gary D. Marty, Kyle A. Garver

**Affiliations:** 1 Fisheries and Oceans, Canada Pacific Biological Station, 3190 Hammond Bay Road, Nanaimo, V9T6N7 Canada; 2 U.S. Department of Agriculture National Coldwater Marine Aquaculture Center, Portage Rd, Orono, ME 04469 USA; 3 Animal Health Centre, Ministry of Agriculture and Food, 1767 Angus Campbell Rd, Abbotsford, V3G2M3 Canada

**Keywords:** Piscine orthoreovirus, Atlantic salmon, Aquaculture, Seawater net-pens

## Abstract

Piscine orthoreovirus genotype-1 (PRV-1) is a virus commonly associated with Atlantic salmon aquaculture with global variability in prevalence and association with disease. From August 2016 to November 2019, 2,070 fish sampled at 64 Atlantic salmon net-pen farm sites during 302 sampling events from British Columbia, Canada, were screened for PRV-1 using real-time qPCR. Nearly all populations became PRV-1 positive within one year of seawater entry irrespective of location, time of stocking, or producer. Cohorts became infected between 100–300 days at sea in > 90% of repeatedly sampled sites and remained infected until harvest (typically 500–700 days at sea). Heart inflammation, which is sometimes attributed to PRV-1, was also assessed in 779 production mortalities from 47 cohorts with known PRV status. Mild heart inflammation was common in mortalities from both PRV + and PRV- populations (67% and 68% prevalence, respectively). Moderate and severe lymphoplasmacytic heart inflammation was rare (11% and 3% prevalence, respectively); however, mainly arose (66 of 77 occurrences) in populations with PRV-1. Detection of PRV-1 RNA was also accomplished in water and sediment for which methods are described. These data cumulatively identify that PRV-1 ubiquitously infects farmed Atlantic salmon in British Columbia during seawater production but only in rare instances correlates with heart inflammation.

**Supplementary Information:**

The online version contains supplementary material available at 10.1186/s12917-022-03409-y.

## Introduction

Piscine orthoreovirus genotype-1 (PRV-1) has commonly been detected among net-pen farmed Atlantic salmon of British Columbia, Canada for more than a decade [1]. Transfer of PRV-1 infected fish from freshwater hatcheries has likely accounted for at least some historical seawater detections owing to the long-term persistence of the virus in infected individuals and the historic identification of PRV-1 at some freshwater facilities [2–4]. Nevertheless, PRV-1 detection in commercial Atlantic salmon freshwater facilities has declined in recent years despite increased screening [5] and PRV-1 has not been reliably detected during Fisheries and Oceans Canada audits of British Columbia freshwater Atlantic salmon facilities since 2018 (Polinski, unpublished data).

Most PRV-1 infections at commercial Atlantic salmon net-pen farms in western Canada have been hypothesized to be acquired at sea [1]. In one temporal study of PRV-1 at a single farm site in 2013, the virus was first detected only after 3 to 4 months of seawater rearing [6]. A second study in 2017 also identified PRV-1 at a farm site after fish had spent 3 months at sea, but not in the same cohort continually held in freshwater [7]. Further, a survey of dead or dying fish collected from net-pen farms in all aquaculture zones of British Columbia from 2011–2013 demonstrated that time-at-sea was a significant predictor of PRV-1 occurrence [8], suggesting a substantial infection pressure from seawater environs in those years. Unclear, however, are the seawater reservoir(s) for PRV-1, or how elimination of freshwater production sources over the past few years has altered the PRV-1 landscape in net-pen farmed Atlantic salmon.

In Norway and Chile, PRV-1 has been identified as an etiological factor for the development of a disease known as heart and skeletal muscle inflammation (HSMI) which has presented an industry production concern for nearly two decades [9–11]. In British Columbia, PRV-1 appears far less associated with disease [2, 7, 12]. Differences in viral genotype can at least partially explain this phenomenon [13] which are also almost certainly further impacted by host and environmental factors [1]. Nevertheless, several instances of HSMI-like heart inflammation have been identified on Atlantic salmon farms in British Columbia [6, 7]. In both instances, affected populations were PRV-1 positive; yet it is unclear as to whether PRV-1’s presence was tangential or integral to these HSMI-like occurrences [3, 7]. Disease causation has been confounded in British Columbia in part by a lack of disease transmissibility [2, 7, 12] and in part by the fact that occasionally Atlantic salmon with HSMI-like heart inflammation have been identified without detectable PRV-1 infections [14].

In this study, we set out to define the current prevalence of PRV-1 across the BC Atlantic salmon farming industry, the putative reservoir (freshwater or marine) contributing to current net-pen infections, and if geographical location is suggested to have contributed to variations in prevalence, source of infection, or disease outcome. This included identifying the length of PRV-1 persistence and load dynamics in infected farmed populations as well as PRV-1’s potential contribution to heart inflammation during Atlantic salmon seawater production. Lastly, we sought to identify if screening of PRV-1 genetic material from environmental sources – specifically seawater and sediment – could be used for identifying PRV-1’s regional occurrence and if such methods could inform on PRV-1’s potential transmission mechanisms.

## Materials and methods

### Sample collection

**Fig. 1:**
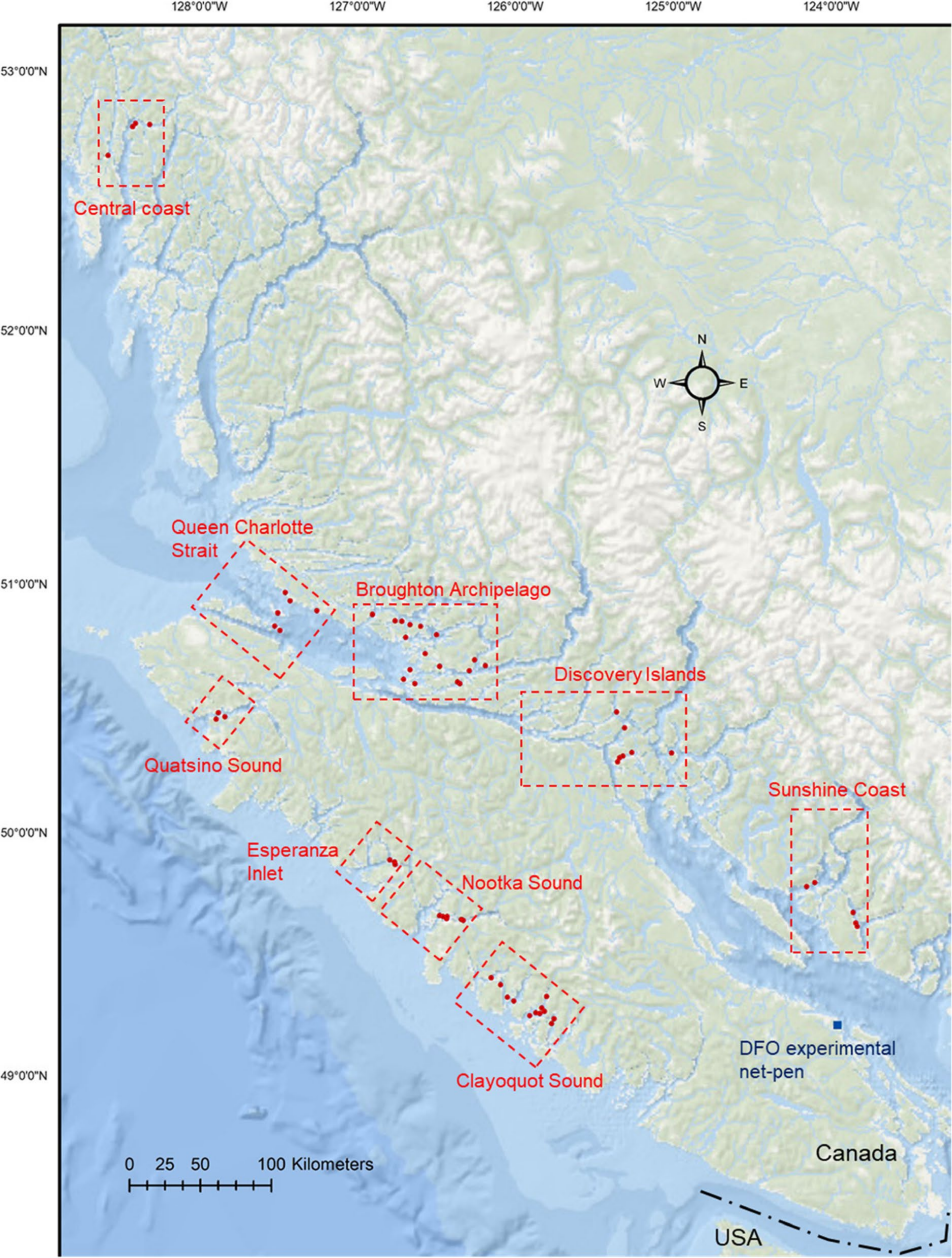
Study Map. Map of study region with indication of commercial (red circles) and DFO research (blue square) net-pen sites sampled in this study. Regional boundaries (dotted red lines) are also provided

Between August 2016 and November 2019, 425 sampling events were conducted opportunistically (based on availability and accessibility) at 64 Atlantic salmon net-pen productions sites in British Columbia, Canada. We grouped the sites into 9 geographically distinct regions to encompass groups of farm sites that were within 10 Nautical miles (NM) of one other, while regional boundaries have at least 15 NM (often larger) between the closest sites in adjacent regions (Fig. 1; Additional file 1).

Fish tissues were collected as part of 302 of the 425 total sampling events from all three commercial Atlantic salmon producers operating in the study region. Typically, 3–20 fish were collected per sampling event and tissues from each fish were screened for the presence of PRV-1 RNA. Seawater was collected as part of 151 sampling events (typically 1–2 samples per event) and benthic sediment was collected as part of 3 sampling events (3–4 samples collected per event) which were also screened for PRV-1 RNA. All seawater and benthic samples were collected from sites operated by a single producer (designated Producer A in this study). Organs were sampled for histopathology as part of 169 sampling events; these included sites operated by all three producers and was used to identify heart inflammation in populations with known or deducible PRV-1 positive or negative status (i.e., individuals from populations testing or previously testing positive for PRV-1 were presumed positive, and individuals from populations at or prior to testing negative were presumed negative). A detailed inventory of all sampling events is provided in Additional file 1. All sampling was conducted under the supervision of an industry fish health manager and/or veterinarian consistent with animal welfare practices of Canadian salmon aquaculture.

In addition to commercial site visits, samples were also collected as part of an experimental ocean net-pen study conducted between June, 2018 and February, 2019 at the Fisheries and Ocean Canada (DFO) Pacific Biological Station (49.2079° N, 123.9599° W) as previously described [15]. During this experimental trial, blood was collected from cohorts of Atlantic salmon held in either duplicate 4,300 L tanks of UV-sterilized seawater, duplicate 4,300 L tanks of raw seawater sourced from Departure Bay, or in a 100 m^3^ experimental net-pen site approximately 300 m offshore in Departure Bay [15]. The experimental net-pen site had been devoid of cultured Atlantic salmon for more than 10 years and is geographically separated from the nearest commercial Atlantic salmon net-pen by more than 37 nautical miles (> 70 km). This provided an opportunity to identify putative non-aquaculture related PRV-1 transmission within a 9-month time frame (Fig. 1). Blood samples were collected at 28-day intervals (*n* = 20 per population per time point) for a period of 252 days and screened for the presence of PRV-1. Animal care and sample collection in the DFO experimental systems was performed in strict accordance with the recommendations set out by the Canadian Council on Animal Care (CCAC) guide to the care and use of experimental animals and all live animal protocols were approved by the Pacific region animal care committee (animal use protocol number: 18–010).

### Blood and tissue collection

All specimens of Atlantic salmon were collected as either recently deceased commercial net-pen mortalities or as live fish dip-netted directly from the commercial or experimental populations. Netted fish were euthanized by a percussive blow to the head and fresh mortalities (denoted by the industry as ‘silvers’) were selected based on gill pallor indicative of death having occurred less than 12 h prior to collection. In all instances, either blood, heart or a combination of spleen, heart and head kidney were aseptically removed for the purpose of PRV-1 molecular screening. Sample type was variable between sites and years; however, in all instances, samples were collected and processed similarly. Blood (100–1000 µL) was obtained from a caudal puncture using a 1 mL syringe and 22-gauge needle and transferred to a 1.5 mL microtube on ice and frozen at -80 °C within 4–6 h. Internal organ samples (50–100 mg) were aseptically excised and preserved in either 1 mL of 70% ethanol or 1 mL of RNAlater™ storage solution (Sigma Aldrich) and frozen at -80 °C within 4–6 h. For histopathology, samples of heart and skin/skeletal muscle (including the lateral line) were preserved directly in 10% Neutral Buffered Formalin and stored at room temperature.

### Seawater and benthic sediment collection

Seawater sampled from within Atlantic salmon net-pen farm sites were collected over the top 10 m using a Watermark® 2.2 L vertical polycarbonate water sampler. Immediately upon collection, the seawater was transferred into a sterile 1 L Nalgene bottle. Either a 250 uL subsample was transferred to a 2 mL sterile screwcap tube or the entire 1 L Nalgene was placed on ice depending on transportation and logistic limitations, and taken to the laboratory where it was frozen at -80 °C until further processing. Benthic sediment samples were acquired from underneath the net-pens using a grab sampler. Upon retrieval, sediment samples were transferred to sterile 50 mL tubes and kept on ice until being placed at -80 °C upon receipt at the laboratory.

### PRV-1 RNA detection

#### Blood and tissue screening

PRV-1 L1 RNA was detected in blood, heart or pooled spleen, heart and head kidney samples by real-time quantitative PCR (qPCR) at one of four diagnostic laboratories. The Fisheries and Ocean Canada Pacific Biological Station Aquatic Animal Health Research Laboratory (DFO-AAH) in Nanaimo British Columbia screened all samples collected from Producer A. The Washington State University Washington Animal Disease Diagnostic Laboratory (WADDL) in Pulman, Washington, USA screened all samples collected from Producer B. Samples collected from Producer C were screened at either the British Columbia Animal Health Centre (BC-AHC) in Abbottsford, British Columbia, Canada or the British Columbia Center for Aquatic Health Sciences (BC-CAHS) in Campbell River, British Columbia, Canada.

In all instances, laboratory screening was conducted based on previously published methods [7, 16, 17]. Specific to screening conducted at DFO-AAH, total RNA was extracted from 100 μL blood or ~ 50 mg tissues in TRIzol Reagent (Life Technologies) as per manufacturer’s instructions that implemented a 5 mm steel bead and TissueLyser II (Qiagen) operating for 2 min at 25 Hz. A portion of eluted RNA (1.0 μg) was denatured for 5 min at 95 °C, immediately cooled to 4 °C, and reverse-transcribed using a High-Capacity cDNA Reverse Transcription kit (Life Technologies) following the manufacturer’s instructions. Resulting cDNA was used directly as template for qPCR analysis in a StepOne-Plus real-time detection system (Applied Biosystems) using previously described primers and TaqMan probe [16]. Each reaction contained 400 nM primers and 300 nM TaqMan probe, 1X TaqMan Universal Master Mix and 1 μL cDNA template within each 15 μL reaction. Cycling conditions included an initial incubation of 95 °C for 10 min followed by 40 cycles of 95 °C for 10 s and 60 °C for 30 s. Samples were assayed in duplicate and were considered positive if both technical replicates reported a Ct value < 40 cycles, inconclusive if only one technical replicate reported a Ct value < 40 cycles, or negative if both technical replicates failed to fluoresce beyond the preset threshold (∆ Rn 0.01) in 40 cycles. PRV RNA quantification was determined in each positive instance by serial dilution of a 482 bp double-stranded DNA gBLOCK fragment (Integrated DNA Technologies) consisting of sequence targeted by the qPCR primer and probe [2]. A seven-step tenfold dilution series of the gBLOCK fragment spanning a dynamic range of 10–10^7^ target copies per reaction was incorporated in duplicate into each run.

Molecular detection of PRV-1 RNA conducted at WADDL, BC-AHC and BC-CAHS was obtained as a fee-for-service. Reports were provided to DFO scientists distinguishing samples as either positive or negative for PRV-1 RNA as determined by qPCR. Relative target quantities (e.g., Ct or estimated target copy number) were not consistently provided by these laboratories and therefore not considered in this study.

#### Seawater screening

All seawater samples were collected at Producer A sites and screened for the presence of PRV-1 L1 RNA at the DFO-AAH by one of two methods. (1) Frozen water samples were thawed at 4 °C and 0.25 mL was transferred to a 1.5 mL tube containing 0.75 mL of TRIzol LS (Life Technologies) with RNA extracted following the manufacturers protocol and PRV testing completed as described above. To increase sensitivity, a second (2) method was used on some samples which utilized thawing the samples at 4 °C and combining 10 mL of sample with 30 mL of ammonium sulphate preservation solution (4 M ammonium sulphate, 25 mM sodium citrate, 10 mM EDTA; pH 7.5). Samples were then inverted 30-40X to mix and centrifuged at 12,000 × g for 10 min at 4 °C. Supernatant was discarded and the pellet containing viral particles was suspended in 1 mL of TRIzol from which RNA was purified, reverse transcribed, and assessed by qPCR as described above. The efficiency of PRV-1 RNA recovery was verified for both methods via spike-in additions of cesium chloride purified PRV-1 in a four-step tenfold dilution spanning a dynamic range of 10^5^–10^2^ spike-in copies per triplicate seawater sample.

#### Benthic sediment screening

All sediment samples were screened for the presence of PRV L1 RNA at the DFO-AAH. PRV-1 RNA was extracted from approximately 2–4 g of sediment using a RNeasy PowerSoil Total RNA Kit (QIAGEN) following the manufactures instructions and eluted in 50 µL of Solution SR7. Approximately 1 µg of RNA was reverse transcribed and subjected to qPCR analysis as described above. The efficiency of PRV-1 RNA recovery was verified by spike-in additions of small volumes of highly infected Atlantic salmon blood into raw presumptively PRV-free sediment collected from the Nanaimo River estuary in a four-step tenfold dilution series spanning a dynamic range of 10^5^–10^2^ estimated spike-in copies per sample.

### Histopathology

Organs preserved in 10% NBF were routinely embedded into paraffin, sectioned at 3 µm, and stained with H&E for light microscopy [4]. Organs from most fish included heart, liver, head kidney, trunk kidney, spleen, intestinal ceca, mesenteries, brain, gill, and skin/skeletal muscle. Samples were submitted by salmon farm fish health staff for routine histopathology as part of the normal diagnostic caseload of the BC-AHC. During the 3 years that project data were collected, five different pathologists provided diagnostic results; none of the pathologists were told that their diagnoses were part of a research project until > 2 years after the last report was competed. Of the 779 hearts subjected to histopathology, 126 (16%) included PRV PCR results as part of the case; pathologists examined the other 653 hearts while blinded to information about the PRV-1 infection status. For heart histopathology, thirteen indices were scored as either being none/not present (0), mild (1), moderate (2) or severe (3). This included the two inflammatory indices of lymphohistiocytic epicarditis (EPL) and endocarditis (ENL) that are most commonly associated with a diagnosis of HSMI; we combined these to give a cumulative heart inflammatory score (0 = none; 1–2 = mild, 3–4 = moderate, 5–6 = severe). HSMI-like heart inflammation has previously been defined as having cumulative heart scores of 4–6 by this method [7, 14]. Micrographs of heart and skeletal muscle inflammation at each score defined in this study are demonstrated in Zhang et al. [12] and Polinski et al. [7]. Also, for each fish, the lead pathologist had listed the lesions or infectious agents that fit in the summary category “Cause/marker of significant morbidity/death.” For this study, we identified all cases in which the pathologists included inflammatory heart lesions in this summary category.

### Statistical analysis

A Pearson correlation coefficient (r) and associated p-value was calculated for time at sea relative to PRV-1 prevalence for the 302 sampling events that sampled fish tissues. PRV-1 relative blood loads (Ct) were compared between normal, moribund, and mortality Producer A specimens by one-way analysis of variance. Relative PRV-1 loads were compared between heart and blood samples in fish which had both samples taken by a paired t-test. Relative PRV-1 blood loads were compared in individuals with known heart inflammation scores (none, mild, moderate, or severe) by one-way analysis of variance. For individuals with matched heart histopathology and PRV-1 RNA blood load results, heart inflammation severity scores were compared between PRV positive and PRV negative populations by Mann–Whitney U and Kolmogorov Smirnov nonparametric tests to assess relative ranks and cumulative distributions, respectively. All statistical analyses were conducted using Graphpad Prism 9.

## Results

### PRV-1 RNA prevalence

**Fig. 2:**
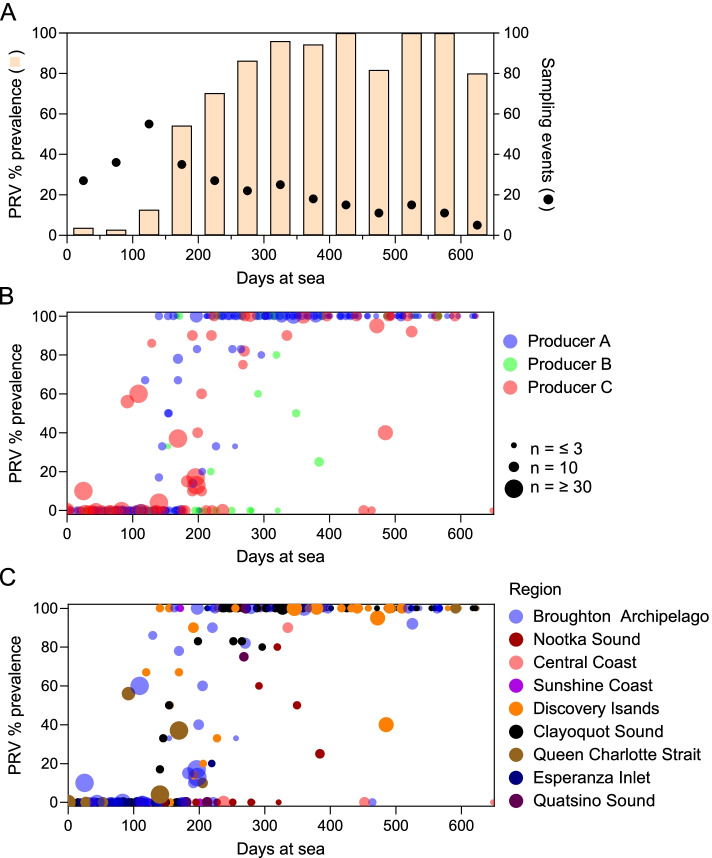
PRV-1 prevalence and distribution. **A** Prevalence of PRV-1 RNA in fish (bar) and number of sampling events (dots) identified for every 50 days at sea. PRV-1 RNA prevalence in relation to days at sea corresponding to (**B**) each of the 3 Atlantic salmon commercial producers in British Columbia as well as (**C**) the 9 geographical regions of production. Sample size (dot size) is indicated in both instances

PRV-1 RNA was identified at 51 of the 64 commercial net-pen farm sites sampled and in all 9 geographical regions in this study (Fig. 1; Fig. 2). Lack of detection in the remaining 13 sites does not indicate that the sites necessarily remained free of PRV-1 during the production cycle, only that the virus was not detected at the time(s) of sampling (Additional file 1).

PRV-1 prevalence was positively correlated with time at sea (*r *= 0.28; 95% CI 0.17–0.38; *p* < 0.0001) (Fig. 2). In the first 90 days following fish stocking into the net-pens, PRV-1 was detected in less than 2% (1/59) of commercial sampling events. In the one event where PRV-1 was detected (in 2018), producer records indicated that PRV-1 had been detected in the freshwater facility late in 2017 where the fish were sourced. For fish populations which had been at sea in net-pens for more than 300 days, 96% (96/100) of sampling events identified PRV-1. Of the 4 instances where PRV was not detected after 300 days at sea, one population (in Nootka Sound) had been sampled 8 times (3–5 fish per time point) in 2018/2019 from 66 to 321 days at sea before the virus was finally detected in 3 of 6 individuals at 349 days at sea. In the second instance, 6/6 fish tested negative at 464 days at sea from a site in the Broughton Archipelago. Interestingly, this population had been transferred from another seawater site in the same region where at 205 days at sea 6 out of 10 individuals tested positive for PRV-1 one week before the transfer. No follow up testing was done at the origin site following transfer. The last 2 instances occurred at sites in the Central Coast region in October of 2017 and July of 2018 where fish had been at sea for 648 and 452 days, respectively. There were no other PRV-1 screenings conducted for these cohorts.

**Fig. 3:**
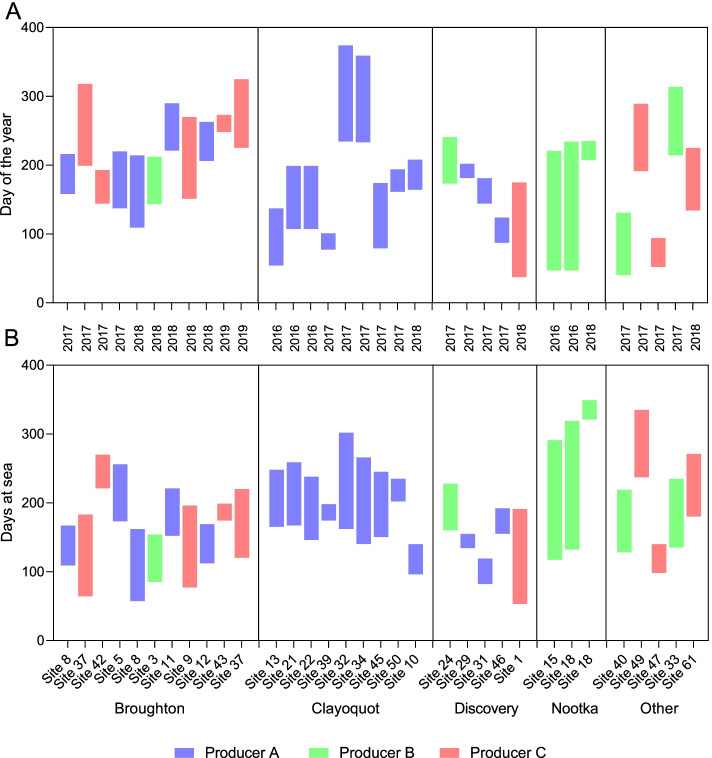
Timing of PRV-1 infection. The number of ordinal days (**A**) or days at sea (**B**) between the last negative and first positive PRV-1 sampling event (colored region) at 30 farm sites across the 9 geographical study regions between 2016 and 2019 where both negative and positive sampling events occurred. The producer (color) as well as the sampling year are indicated for each net-pen site

Nearly all (> 90%) first PRV-1 detections in repeatedly sampled populations occurred between 100–300 days at sea irrespective of year, producer, region, or farm site (Fig. 2; Fig. 3). Sites became infected during almost all months of the year; however, two regions appeared to have relatively consistent seasonality for first detections, albeit at low temporal resolution. In the Broughton Archipelago, first detections occurred only in the latter half (July – December) of the calendar year in the three years of this study (2017, 2018, and 2019) (Fig. 3). In Nookta Sound, first detections occurred only in late August or early September in 2016 and 2019 when sampling was conducted. In contrast, no seasonality for first PRV-1 detection was observed in net-pens in the Clayoquot Sound where first detections occurred in almost all months of the year (Fig. 3).

Among the 26 instances where PRV-1 positive sites were resampled after first detection, PRV-1 was detected in all sites and during all resampling events. Prevalence was usually high (typically 100%) at resampling (Fig. 2). The longest period between first detection and last positive resampling in this study was 460 days.

PRV was not detected in any of the 9 sampling events conducted over the 252-day research study involving tanks and net-pen populations at the DFO research facility in Departure Bay. Both net-pen and raw seawater-fed tank fish did, however, become infected with *Kudoa sp*. and *Piscirickettsia salmonis* during the study as previously reported [15].

### PRV-1 RNA loads in infected individuals

**Fig. 4:**
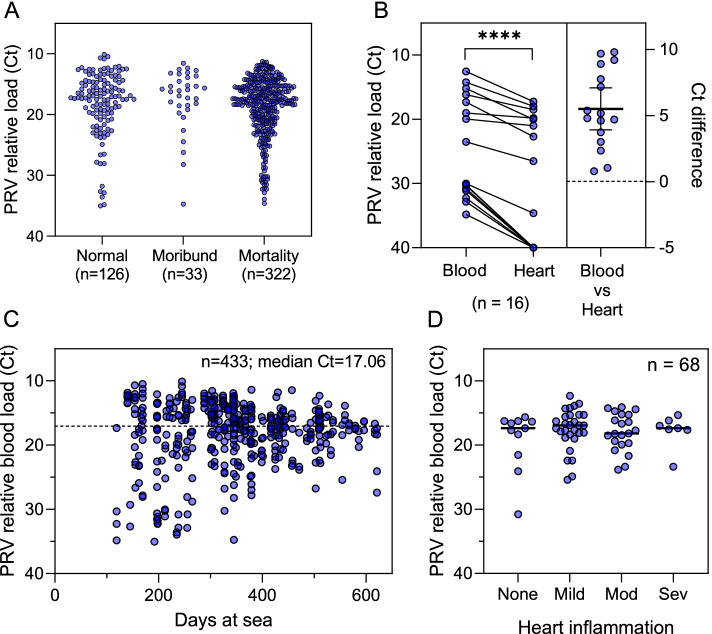
PRV-1 loads. **A** Individual (dot) PRV-1 RNA blood loads collected from either recent mortalities, moribund individuals, or members of the normal Atlantic salmon population across 19 commercial net-pen farms sites in British Columbia are contrasted by one-way ANOVA. **B** qPCR Ct values for blood and heart samples for 16 fish in which both blood and heart samples were simultaneously collected are compared by a paired T-test (**** = *p*-value < 0.0001. **C** the individual distribution (dots) and mean (dotted line) of qPCR Ct from all blood samples from a single commercial producer are presented relative to days at sea. **D** mean (line) and individual (dot) PRV-1 RNA blood loads of fish for which the combined heart histopathology score for lymphohistiocytic epi- and endo-carditis was either not present, mild, moderate (mod), or severe (sev)

The mean relative PRV-1 blood load was similar between individuals from the normal (*n* = 126; mean Ct = 18.1 ± 5.2 SD), moribund (*n* = 33; mean Ct = 17.5 ± 5.0 SD) or mortality (*n* = 322; mean Ct = 18.6 ± 4.8 SD) populations of fish sampled from 19 farm sites operated by Producer A in this study (*p* > 0.3; Fig. 4A). Of the 16 individuals which had both blood and heart samples collected, Ct comparisons showed significantly lower Ct (higher PRV-1 RNA loads) in blood compared to heart samples in these individuals (p < 0.0001) with a mean Ct difference of approximately 5 (Fig. 4B). PRV-1 blood load was variable between individuals and populations in this study with the greatest variance occurring early during the infection processes. However, variance decreased with increased time at sea with an overall mean blood Ct of 17 (Fig. 4C).

### Heart inflammation in relation to PRV-1 infection

**Fig. 5:**
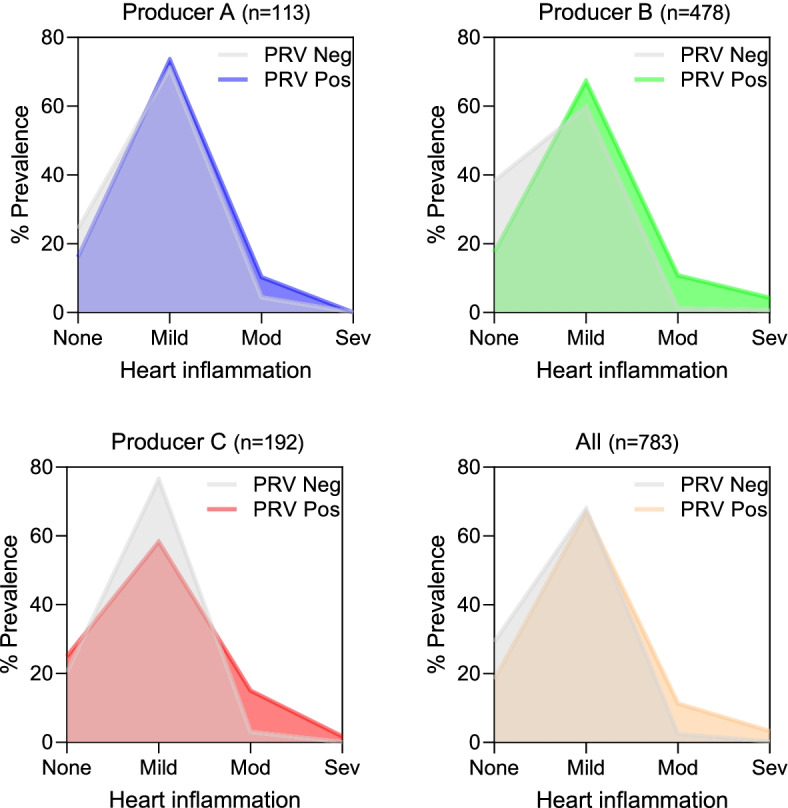
Heart Inflammation relative to PRV-1 status. Prevalence of heart inflammation (combined score for lymphohistiocytic epi- and endo-carditis) in Atlantic salmon net-pen production mortalities across sites from all three regional producers is presented for both PRV-1 positive and PRV-1 negative populations. For Producer A, heart inflammation scores and PRV-1 status were matched to individual fish. For Producers B and C, PRV-1 status is indicated at the population level as fish screened for PRV-1 were not necessarily the same fish screened by histopathology for heart inflammation

There were 113 individual Atlantic salmon mortalities from Producer A collected from 21 net-pens where both PRV-1 blood screening results and matched heart histopathology were available from the same fish. Of these, 45 were PRV- and 68 were PRV + . There was no significant difference by rank (*p* > 0.57) or by cumulative distribution (*p* > 0.98) of EPL + ENL heart pathology scores in PRV + versus PRV- individuals. In the 68 PRV + mortalities, relative viral blood load (Ct) was similar between fish with either mild, moderate, severe, or no heart inflammation (*p* > 0.58; Fig. 4D). Additionally, there were 148 sampling events conducted at producer B and C net-pens for which heart pathology was assessed: 57 events included fish presumed PRV- and 112 events included fish presumed PRV + based on PRV-1 screening of individuals within each cohort (Additional file 1). Cumulatively, mild heart inflammation was common (60–77% prevalence) among net-pen reared salmon mortalities irrespective of PRV status or producer (Fig. 5). Moderate to severe heart inflammation was rare; however, in most instances occurred in PRV + individuals. There were 24 out of a total of 783 production fish (3%) with heart inflammation considered to be HSMI-like (severity scores of 4–6). Of these, 23 were from PRV + populations and 1 was from a PRV- population.

There were only 2 fish in this study which had skeletal muscle inflammation greater than mild (both diagnosed as moderate); neither of these fish had significant heat lesions. Mild skeletal muscle inflammation was, however, more common among the 24 fish with HSMI like heart inflammation (25% with mild skeletal muscle inflammation) than among the overall sampled population (5.6% with mild skeletal muscle inflammation). Among the 14 fish diagnosed with significant heart lesions but without major lymphohystiocytic inflammation (EPL + ENL was < 4), none had skeletal muscle inflammation (Additional file 1).

### PRV-1 RNA detection from environmental samples

**Fig. 6:**
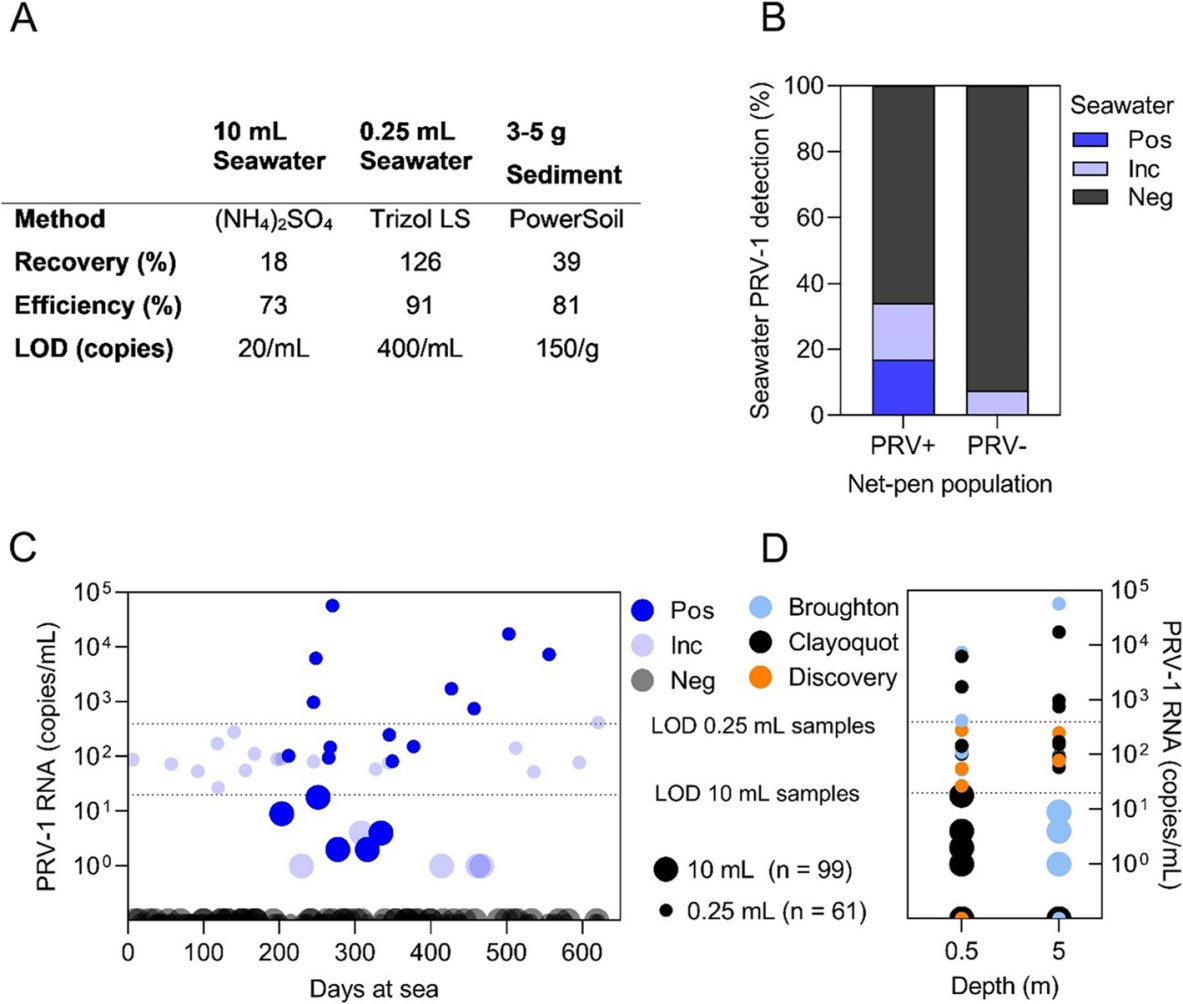
Detection of PRV-1 RNA from water and sediment. **A** RNA recovery using either ammonium sulfate preservation solution [(NH_4_)_2_S0_4_], Trizol LS, or Qiagen Powersoil total RNA kits were employed on either water or sediment samples. Mean percent recovery of PRV-1 spiked into each sample matrix along with relative qPCR efficiency for detection is presented over a dynamic range of 10^5^–10^2^ copies/sample. The limit of detection (LOD) at which qPCR detection occurred in greater than 80% of technical replicates is also indicated. **B** Seawater detection prevalence of PRV positive (Pos; detection in both technical replicates), inconclusive (Inc; 1 of 2 technical replicates with Ct > 32) or PRV negative (Neg; no detection in either technical replicates in 40 PCR cycles) test results are presented for sites with net-pen populations of Atlantic salmon where fish in the population had internal tissues that were positive for PRV-1 RNA (PRV+) or where the virus was not detected (PRV-). **C** PRV-1 RNA estimated copies in PRV positive (Pos; mean copies from two replicates), inconclusive (Inc; copies for the single positive replicate) or PRV negative (Neg; no detection in in 40 PCR cycles) seawater samples that **D** came from sites located in either the Broughton Archipelago, Clayoquot Sound or Discovery Islands region. Seawater sample volumes of either 10 mL or 0.25 mL are indicated by the size of the data point and the LOD as defined by > 80% consistency in detection is indicated with a dotted line for each sample volume

Detection of PRV-1 spiked into water and sediment samples was reliable (limit of detection defined here as > 80% probability of detection) in identifying PRV-1 RNA at concentrations as low as 20 copies/mL seawater and 150 copies/g benthic sediment (Fig. 6A). Recovery from seawater using TRIzol LS was similarly efficient to commonly published recovery methods employing TRIzol on infected fish tissue or cell culture medium [i.e., 90% qPCR efficiency and 100% recovery of spiked material [18]]. Centrifugal concentration of PRV-1 in the presence of ammonium sulfate preservation solution was also effective at detecting PRV-1, and functionally reduced the limit of detection (LOD) by 20X (Fig. 6A). However, mean proportional recovery of spiked virus was reduced to 18% and qPCR efficiency was lower than for standard methods (73 vs ~ 90%), indicating viral recovery using ammonium sulfate precipitation could theoretically be further improved, particularly at low target concentrations. The Qiagen Powersoil total RNA kit also showed moderate loss in proportional recovery of spiked virus from sediment, specifically at low concentrations as evidenced by a reduced qPCR efficiency compared to standard methods (81 vs ~ 90%; Fig. 6A).

Reliable detection of PRV-1 RNA in seawater samples only occurred at sites where fish populations were PRV+ , and this pattern was consistent in all 3 geographical regions where water sampling was conducted (Fig. 6B, 6D). Also, inconclusive PRV-1 detections occurred at sites with both PRV+ and PRV- populations and in all geographic regions. The majority (70%) of water samplings did not detect PRV-1 RNA irrespective of the infection status of the site population. In most instances, positive or inconclusive detections also occurred at or below the reliable limit of detection for the qPCR assay (Fig. 6C).

PRV-1 RNA was detected in all 12 sediment samples collected during all 3 sampling events at 2 commercial production sites. This included samples collected at a site in the Discovery Islands which had been fallow for approximately 3 months at the time of sampling, as well as at a site in the Broughton Archipelago before and after fish on the site became infected with PRV-1 (Table 1).

**Table 1:** Summary of sediment screening results and associated net-pen information. Three or 4 sediment samples were collected at each site-specific sampling event

Region	Site	Sample date	Site status	Sediment result	Mean Ct
Discovery	29	4/9/2019	Fallowed over 2 months when sampled; 1 week prior to fish stocking	Pos	33.07
				Pos	31.42
				Pos	30.57
				Pos	29.56
Broughton	12	2/25/2018	Site stocked 112 days; fish PRV- (0/15) on day of sampling	Pos	28.87
				Pos	31.27
				Pos	29.96
				Pos	27.99
Broughton	12	10/24/2018	Site stocked 203 days; fish PRV + (15/15) 6 days prior to sampling	Pos	28.45
				Pos	28.95
				Pos	30.00

## Discussion

The hypothesis that most PRV-1 infections of commercially produced Atlantic salmon in Western Canada are acquired at sea is confirmed for the period encompassed by this study (2016–2019). The identification of seawater reservoirs of PRV-1 in all farming areas of Western Canada also verifies the widespread prevalence of the virus in the region [1]. Although putative historical contributions to PRV-1 prevalence from commercial freshwater facilities are unknown, it is now clear that PRV-1 can and does perpetuate in coastal environments of British Columbia independent of commercial freshwater introductions. This implies that farmers currently have little control over if or when their fish become infected by PRV-1 once they are stocked to net-pens.

As salmon appear to be the primary (if not exclusive) long-term reservoir of PRV-1 in the Northeastern Pacific [1], it is logical to assume that the seawater reservoir of PRV-1 is made up of commercial salmon stocks, wild stocks, or both. We use data from this study to hypothesize that both are likely. In arguing for commercial net-pen populations as a regional reservoir, we identified that nearly all net-pen sites have this potential given that nearly all showed evidence of becoming PRV infected and maintained these infections for sometimes well over a year. Although specific shedding and putative transmission from net-pens is unknown, previous experimental transmission studies [2, 7] coupled with the environmental seawater detection of viral nucleic acid in this study provides strong evidence for the potential that shedding of viable virus from net-pens transiently occurs. Additionally, data from this study indicate that in at least some regions such as the Clayoquot Sound – where PRV+ populations are present year-round, farms are within a few nautical miles of one another, and first detections occur in almost every month of the year – farm-to-farm transmission presents a likely contributor to PRV prevalence on farms in the region.

In other regions, an argument for a wild fish reservoir is better suited. For example, in Nootka Sound – where the region is fallowed for 2–3 months between production cycles, the nearest positive site is in an adjacent region many nautical miles away, and first detections appear to occur seasonally and only after 10 or more months at sea – the source of infection seems likely to be from a wild migrating population of Chinook or coho salmon in the sound. This is supported by the fact that PRV-1 is common in both Chinook and coho species of British Columbia [1] and that transmission between (and therefore from) wild fish occurs independent of farms in the northeastern Pacific as evidenced by PRV-1 detection in populations in Alaska and southern Washington state where fish farming does not occur [19]. We therefore speculate that for most sites in this study area, both adjacent farms (where present) and wild salmon have the potential to act as an infection source of naive net-pen stocks and combine to form a regionally ubiquitous seawater reservoir for PRV-1 in coastal British Columbia.

Following experimental infection, PRV-1 has shown a tropism for red blood cells and, to a lesser extent, heart tissues, with no evidence of viral clearing in either sample type [2, 20]. Our study confirms a blood tropism by PRV-1 during commercial net-pen production with little evidence that the virus is cleared from an infected population. Indeed, PRV-1 was detected in every sampling event at each of the 26 PRV+ sites where repeated sampling took place. The only putative evidence for viral clearing in this study was when PRV-1 was detected in 60% of samples at a Broughton site but not at a second Broughton site 200+ days later after a fish transfer had occurred. Although this could represent the first putative evidence for clearing of PRV-1 from an infected population, it could also be possible that PRV+ fish were missed in the random sample or that testing produced a false-negative result. Currently, cumulative evidence supports that PRV-1 is not cleared from a population of farmed Atlantic salmon once infected.

This study also confirms that for maximizing early detection, screening of blood relative to heart is recommended. During late-stage infection, either tissue is acceptable due to the systemically high viral titers which are maintained in both sample types. These data in conjunction with data from previous laboratory challenge trials indicate that relative quantity can be utilized to give a rough dichotomous (early vs late) estimation of the infection stage and thereby the timing of infection. Specifically, low relative quantities (for example, blood Ct values > 25 in this study) suggest that the fish was recently infected – likely within the past few weeks – whereas high relative quantities (i.e., blood Ct values < 20 in this study) suggest that individuals have probably been infected for a considerable time – i.e., a month or more [7, 12]. In addition, this study confirms that relative PRV-1 load was not useful as a predictor of morbidity or mortality in a commercial net-pen setting which is similar to results of field study [3] and previous laboratory challenge trials [7]. This reiterates that PRV-1 load cannot be used as a disease proxy.

The Fish Health Auditing and Surveillance Program (FHASP) conducted by DFO Aquaculture Management Division previously evaluated heart tissues of nearly 6,000 Atlantic salmon net-pen farming mortalities from 2006–2018 and found 61% prevalence of mild to moderate heart inflammation [21]. We identified a similar percentage (68%) of mild heart inflammation in production mortalities in this study during an overlapping period (2016–2019). We further identified that this condition occurred equally in PRV + and PRV- populations as well as PRV+ and PRV- individuals, indicating that PRV-1’s contribution to the prevalence of mild heart inflammation has been minimal to non-existent during net-pen culture in Western Canada.

The 2006–2018 FHASP also identified heart inflammation with a severity sufficient to be a putative cause or contributing factor to death in approximately 3% of sampled mortalities, which is similar to what we identified here (2.4% with heart lymphohistiocytic inflammation histopathology scores of 4–6, and 4.2% of cases in which inflammatory heart lesions were diagnosed as contributing to morbidity).This is consistent with historical observations from the 1990’s [22, 23]. We were able to further identify that the supermajority (23 of 24) of these HSMI-like occurrences happened in populations that were infected with PRV-1, suggesting that PRV-1 was a possible contributing factor to this condition. It was also noteworthy that mild skeletal muscle inflammation was nearly 5 times more prevalent among the 24 fish with HSMI-like heart inflammation relative to the total population (25% vs 5.6%, respectively). Nevertheless, idiopathic and disease associated net-pen mortalities in recent years have accounted for approximately 7% of the average production cohort [21]. Thus, PRV-1 associated heart inflammation (occasionally concurring with mild skeletal muscle inflammation) appears to have contributed to or caused approximately 0.2% - 0.3% mortality (i.e., 2.4 – 4.2% of 7%) in net-pen cultured Atlantic salmon of British Columbia during 20-month production cycles in the last few years, and likely has done so for the past 25 years. Noteworthy, however, is that 1/24 (4%) of observed HSMI-like heart inflammation in this study occurred in the absence of PRV-1, which has also been observed previously [14]. This indicates that at least in British Columbia, there are other factors independent of PRV-1 which can cause HSMI-like heart inflammation in farmed Atlantic salmon. The pathologists in our study diagnosed another 14 cases in which other types of heart inflammation contributed to significant morbidity, and all of these fish were from PRV + populations. These other cases provide examples of the range of heart lesions that occur among farmed Atlantic salmon, but the role of PRV in these cases, if any, is not known.

Environmental detection of PRV-1 nucleic acids has potential utility for expanding early detection when prevalence is low or increasing sensitivity to include material associated with non-resident hosts. Environmental RNA in marine systems has recently been shown to be unexpectedly stable, particularly when associated with biofilms [24] which may be even further extended by protection from an intact viral capsid or subviral particle. In this study, PRV-1 was consistently identified from replicate sediment samples at an active farm both before and after the virus was detectable in fish as well as from a farm more than two months after infected fish had been removed. This suggests that either PRV-1 genetic material is environmentally stable in sediment for multiple months, or that a secondary local host reservoir is present at these locations. Further investigation will be needed to determine if either of these scenarios is valid, although we speculate prolonged environmental stability is more likely. It is also uncertain if this sediment-associated virus contains infectious particles, and if so, if they can act as an infection source for salmon held in net-pens meters above.

By concentrating virus via ammonium sulphate-based precipitation, we were able to reliably detect PRV-1 in seawater to as low as 20 copies/mL (2 × 10^4^ copies/L). In some instances, PRV-1 was identified at or above these concentrations around infected farms, suggesting that genetic material was being shed by infected farmed fish. Inconclusive detections also repeatedly occurred for which concentrations (unsurprisingly) were below the effective limit of detection of our assay. Although some of these detections could represent false positive detections, it is also likely that at least some of these detections represent true positive samples – it is currently impossible to distinguish one from the other. Thus, using our current methodology, seawater detection may be effective at identifying highly infected populations, but may be insensitive as an alternative to live fish sampling due to an inability to differentiate infectious vs non-infectious material, and it is not able to differentiate the source of the detection (e.g., farmed fish vs. wild fish in the same area).

In conclusion, this study confirms the regionally widespread seawater occurrence of PRV-1 in net-pen reared Atlantic salmon of British Columbia with most farms becoming infected within 100–300 days of stocking. Mild heart inflammation – which was prevalent in farmed Atlantic salmon of British Columbia irrespective of PRV-1 during this study period – developed into moderate or severe heart inflammation in rare instances which typically manifested in the presence of PRV-1. PRV-1 contributions to these rare cases of heart inflammation appear likely, however the mechanism and secondary requirements associated with such disease development remains unclear. Timing of infection, which was sometimes unknown or imprecise in this study, is also likely important in defining PRV-associated heart inflammation and will be important in further clarifying PRV’s putative role in disease. Environmental detection of virus was also demonstrated at farm sites but provided minimal insight into PRV-1 prevalence and transmission potential within the region beyond what could be deduced from fish sampling.

## Supplementary Information

**Additional file 1.** Excel file of collected and generated data including sample event inventory, PRV-1 qPCR screening results, histopathological heart scoring, and comparative heart inflammation/PRV-1 assessments.

## Data Availability

All data supporting the conclusions of this article are available in the supplementary information associated with this publication as well as in Figshare digital repository, https://doi.org/10.6084/m9.figshare.19759195.
